# Food allergen sensitization patterns in Korean adult food allergy patients

**DOI:** 10.1186/1939-4551-8-S1-A7

**Published:** 2015-04-08

**Authors:** Sungryeol Kim, Kyung Hee Park, Jung-Won Park, Jae-Hyun Lee, Hye Jung Park, Beom Seok Koh, Il Joo Moon, Dong Woo Leem

**Affiliations:** 1 Yonsei University College of Medicine, South Korea

## Background

Identification of the causative food in food allergy patients is crucial. However, offending allergens can vary with a country’s food choices and preparation methods. In this study, we analyzed the sensitization rate to specific food allergens in Korean adult food allergy patients.

## Methods

This study enrolled 134 adult patients who visited the allergy clinic of Severance Hospital due to their allergic symptoms related to food ingestion. Patients underwent skin prick test (SPT) with 55 allergens. Our food SPT panel included hairtail, yellow corvina, common eel, skate, squid, mackerel, anchovy, saury, octopus, chrysalis, sunflower seed, and pollock allergens prepared at our Institute of Allergy and reflecting the daily eating habits of Korean people.

## Results

Of the 134 patients, 73 (54.5%) were sensitized to one or more food allergens. The sensitization rate of men (69.2%) was higher than that of women (45.1%) (*p* = 0.008).

Sensitization to chrysalis was detected most frequently at a rate of 25.4%. Sensitization rates to other food allergens prepared by us or that were relatively highly sensitized were as follows: maize grain (13.4%), shrimp (11.9%), almond (11.1%), sunflower seed (8.2%), mackerel (5.2%), pollack (5.2%), halibut (4.5%), anchovy (4.4%), squid (3.7%), saury (3.0%), common eel (3.0%), yellow corvina (3.0%), hairtail (2.2%), octopus (2.2%), and skate (0.7%).

## Conclusions

Food sensitization patterns in Korean food allergy patients are different from those in other countries. Chrysalis showed the highest sensitization rate in Korean patients (25.4%). Interestingly, mackerel, pollack, halibut, anchovy and yellow corvina which are popular food ingredients in Korea were also highly sensitized. Therefore skin prick test panel is need to reflect the preferred food choices of a region.

